# *Candida auris* persists in the vaginal microaerobic niche in the absence of interleukin-17A

**DOI:** 10.1128/msphere.00446-25

**Published:** 2025-10-08

**Authors:** Masahiro Abe, Sota Sadamoto, Akiko Nagamori, Minoru Shinozaki, Sayoko Oiki, Amato Otani, Ami Koizumi, Takayuki Shinohara, Yoichiro Iwakura, Kazutoshi Shibuya, Yoshitsugu Miyazaki

**Affiliations:** 1 Department of Fungal Infection, National Institute of Infectious Diseases, Japan Institute for Health Security, Tokyo, Japan; 2 Department of Surgical Pathology, Toho University School of Medicine, Tokyo, Japan; 3 Division of Experimental Animal Immunology, Research Institute for Biomedical Sciences, Tokyo University of Science 13170 , Chiba, Japan; 4 Louis Pasteur Center for Medical Research 89270, Kyoto, Japan; 5 Department of Pathophysiology and Infection Control of Fungal infection, Toho University School of Medicine, Tokyo, Japan; University of Michigan Michigan Medicine, Ann Arbor, Michigan, USA

**Keywords:** vulvovaginal candidiasis, *Candida auris*, interleukin-17A, neutrophils, S100A8

## Abstract

*Candida auris* is an emerging fungus notable for its high drug resistance and persistent colonization of human hosts and environmental surfaces. However, its role in vulvovaginal candidiasis (VVC), a common form of superficial candidiasis, remains poorly understood. In this study, we investigated the colonization capacity of *C. auris* and vaginal defense mechanisms in a VVC model. Using an estrogenized VVC mouse model, we evaluated fungal burden, inflammatory cell counts, and S100A8 concentrations in vaginal lavages of wild-type (WT) and IL-17A knockout (*Il17a^−/−^*) C57BL/6J mice following *C. auris* inoculation. Histopathological examination and flow cytometry analysis of vaginal immune cells were also conducted. Additionally, an *in vitro* adhesion assay was performed using VK2/E6E7 vaginal epithelial cells under aerobic and microaerobic conditions mimicking the vaginal environment. Persistent colonization by *C. auris,* particularly clades I, III, and IV, with minimal infiltration of inflammatory cells, was confirmed in *Il17a^−/−^* mice. These findings were also supported by histopathological analysis. S100A8 concentration analysis revealed significant differences between WT and *Il17a^−/−^* mice, with lower levels detected in the *Il17a^−/−^* group. Furthermore, S100A8 levels showed positive correlations with inflammatory cell count and negative correlations with vaginal fungal burden. Flow cytometry analysis demonstrated a reduced number of vaginal neutrophils in *Il17a^−/−^* mice. Additionally, *in vitro* adhesion assay revealed increased *C. auris* adherence to vaginal epithelial cells under microaerobic conditions. *C. auris* exhibits a strong affinity for the vaginal epithelium, and IL-17A appears to play a protective role in *C. auris-*associated VVC.

**IMPORTANCE:**

*Candida auris* is an emerging fungal species, and several reports have recently identified C. auris in patients with vulvovaginal candidiasis (VVC), although few studies have investigated the relationship between C. auris and VVC or the associated host factors. Our study, using the VVC mouse model, confirmed persistent vaginal colonization by *C. auris*, especially clades I, III, and IV, along with reduced neutrophil infiltration and lower S100A8 secretion under interleukin-17A-deficient conditions. In addition, *in vitro* assays demonstrated enhanced *C. auris* adhesion to vaginal epithelial cells, especially microaerobic conditions imitating human vaginal microenvironments. Our findings suggest that *C. auris* exhibits strong vaginal tropism, and IL-17A plays a critical role in controlling *C. auris*-associated VVC.

## INTRODUCTION

*Candida* species are opportunistic fungi that colonize the skin and mucosal membranes, occasionally leading to superficial infections. Vulvovaginal candidiasis (VVC) is one of the most common types of superficial candidiasis, affecting approximately 75% of women at least once during their lifetime (1–6). *Candida albicans* is the predominant cause of VVC, accounting for 60%–90% of vaginal *Candida* isolates, although all *Candida* species can act as causative pathogens (2, 7–10). *Candida auris*, originally isolated from patients with chronic otitis media, is an emerging fungal species that has become a global health concern due to its high antifungal resistance, prolonged colonization in humans and the environment, and its potential to cause nosocomial bloodstream infection outbreaks (11–15). Recently, *C. auris* has been identified as a cause of VVC in a few case reports, although the incidence of *C. auris* VVC is quite low, and this disease is not consistently seen in clinical settings (16–18). Moreover, it has been reported that *C. auris* can induce vaginal inflammation and epithelial damage (19). Nevertheless, few studies have investigated the relationship between the emerging fungus *C. auris* and VVC or the associated host factors.

Interleukin-17A (IL-17A) has been implicated in the immune response against superficial candidiasis. Defects in, or inhibition of, the IL-17 pathway have been closely associated with oropharyngeal, esophageal, and cutaneous candidiasis (20–23). In the context of VVC, however, the role of IL-17A remains controversial. While some studies suggest a protective function of IL-17A against *C. albicans* vaginal infections, others have reported that IL-17 signaling is dispensable in VVC (24–27). Notably, most previous research has focused solely on *C. albicans*, and studies on non-albicans *Candida* species in superficial infections remain limited. With regard to *C. auris*, prior research has demonstrated a link between IL-17 signaling and *C. auris* cutaneous colonization (28–30), suggesting that IL-17 may also play a role in *C. auris* VVC.

In this study, we investigated the relationship between vaginal *Candida auris* infections and IL-17A using a mouse estrogen-dependent pseudo-estrus VVC model. Specifically, we assessed vaginal fungal burden, inflammatory cell accumulation in vaginal lavages, and secretion of the antimicrobial peptide S100A8, comparing wild-type (WT) mice with IL-17A-deficient (*Il17a^−/−^*) mice. Additionally, we conducted histopathological analysis of vaginal tissues, evaluated vaginal immune cells via flow cytometry, and examined the *in vitro* adhesive capacity of *C. auris* to vaginal epithelial cells under microaerobic conditions to further elucidate the pathogenesis of *C. auris* VVC.

## RESULTS

### Vaginal persistent fungal colonization in *Il17a^−/−^* mice

**Fig 1:**
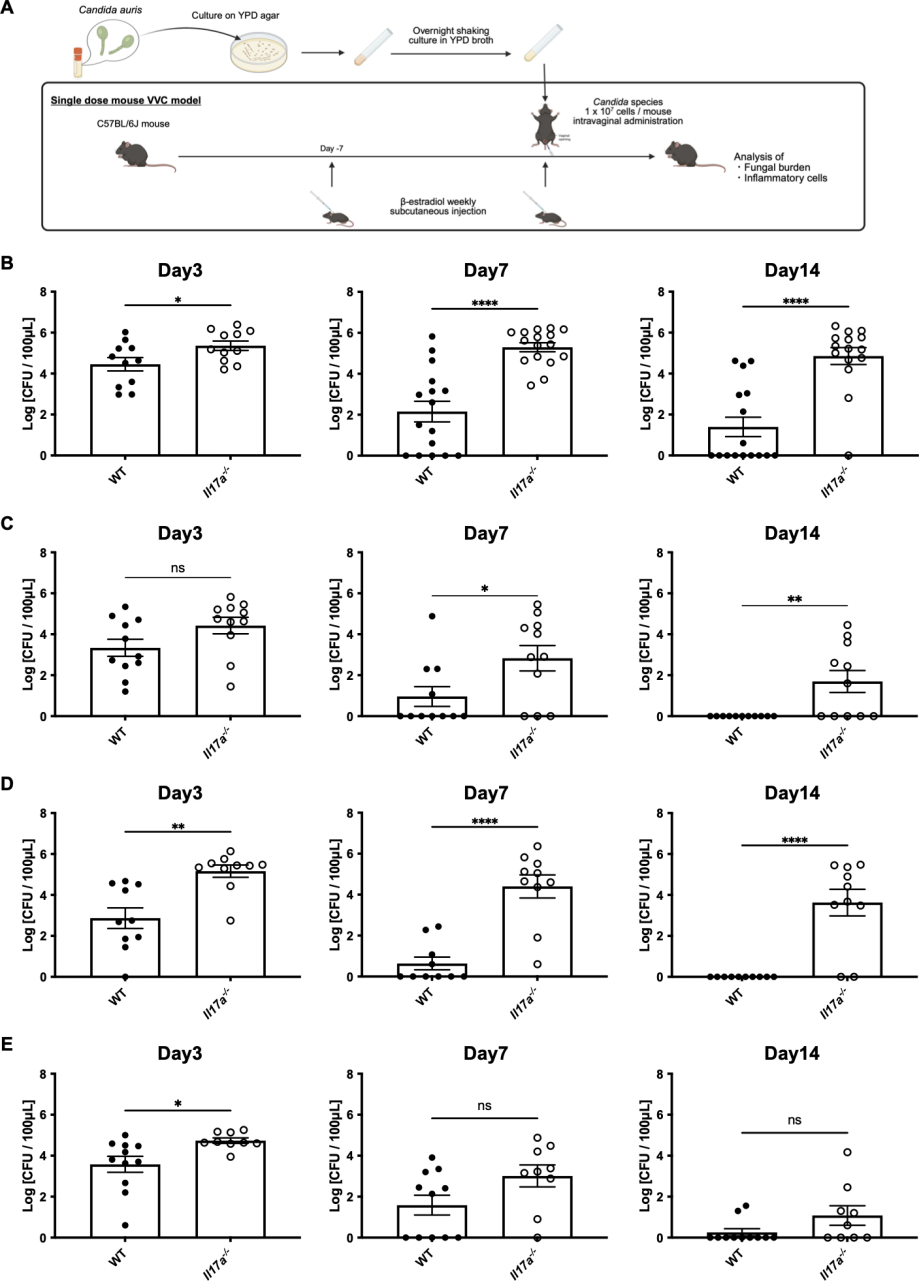
(**A**) The scheme of the *in vivo* vulvovaginal candidiasis model is shown. The burden of *C. auris* AR 0382 (**B**), AR 0383 (**C**), AR 0385 (**D**), and AR 0381 (**E**) in the vaginal lavage after inoculation. Fungal burdens in the vaginal lavage are shown as log (CFU/100 µL). All results are from at least three independent experiments and are expressed as mean ± standard error of the mean. ns, not significant; *P < 0.05, **P < 0.01, ****P < 0.0001.

We evaluated the vaginal fungal burden of *C. auris* following intravaginal inoculation and compared the results between WT and *Il17a^−/−^* mice using the VVC model (Fig. 1A). Three days post-inoculation, the vaginal fungal burden of *C. auris* AR 0382 and AR 0385 was significantly higher in *Il17a^−/−^* mice compared to WT mice. Furthermore, consistent colonization was observed until the terminal time point of day 14 post-infection in *Il17a^−/−^* mice, whereas the fungal burden in WT mice gradually declined over time (Fig. 1B through D). In contrast, strain AR 0381 showed a decreasing fungal burden in both WT and *Il17a^−/−^* mice, with no statistically significant differences observed (Fig. 1E). These results suggest that *C. auris*, particularly clades other than clade II, can establish persistent vaginal colonization under IL-17A-deficient conditions.

### Fewer vaginal inflammatory cell infiltration in *Il17a^−/−^* mice

**Fig 2:**
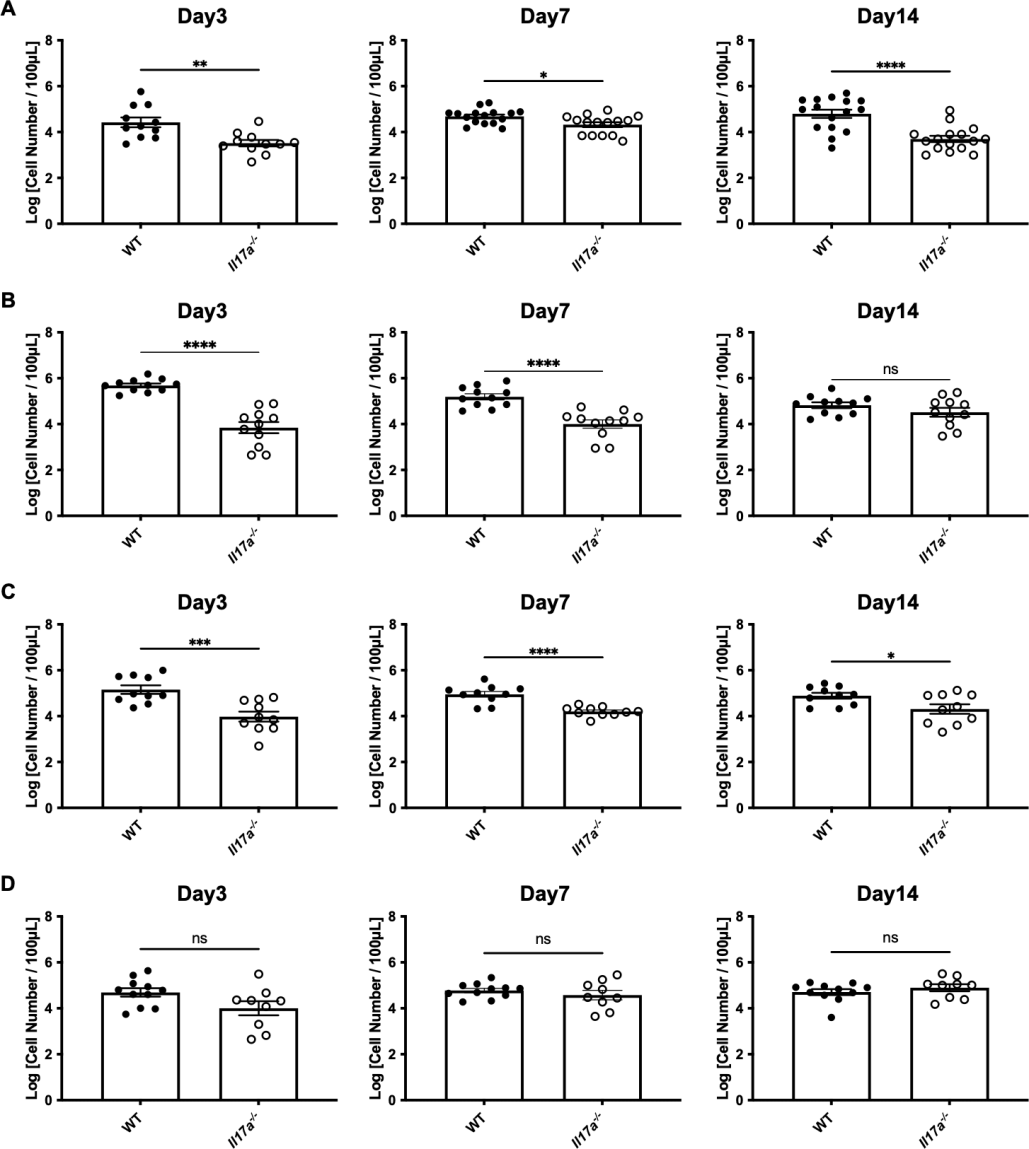
The number of inflammatory cells after inoculation with *C. auris* AR 0382 (**A**), AR 0383 (**B**), AR 0385 (**C**), and AR 0381 (**D**) in the vaginal lavage. The number of inflammatory cells in the vaginal lavage is shown as log (cell number/100 µL). All results are from at least three independent experiments and are expressed as mean ± standard error of the mean. ns, not significant; *P < 0.05, **P < 0.01, ***P < 0.001, ****P < 0.0001.

We also evaluated the infiltration of inflammatory cells in vaginal lavages by counting viable cells using trypan blue staining and a hemocytometer, based on cell morphology alone. In contrast to the trend observed for vaginal fungal burden, the number of inflammatory cells in WT mice was significantly higher than in *Il17a^−/−^* mice three days after inoculation with AR 0382, AR 0383, or AR 0385 (Fig. 2A through C). In addition, most of the inflammatory cells in vaginal lavages were identified as neutrophils by flow cytometry analysis (Fig. S1). These significant differences in vaginal inflammatory cell numbers between WT and *Il17a^−/−^* mice persisted up to 14 days after *C. auris* inoculation, although the absolute differences between the two groups became smaller over time (Fig. 2A through C). In contrast, for the *C. auris* clade II isolate AR 0381, there were no significant differences between WT and *Il17a^−/−^* mice throughout the experiments (Fig. 2D). Collectively, these data suggest that clade I, III, and IV *C. auris* isolates can induce inflammatory cell infiltration into the vagina in an IL-17A-dependent manner.

### Less secretion of antimicrobial peptide S100A8 in *Il17a^−/−^* mice

**Fig 3:**
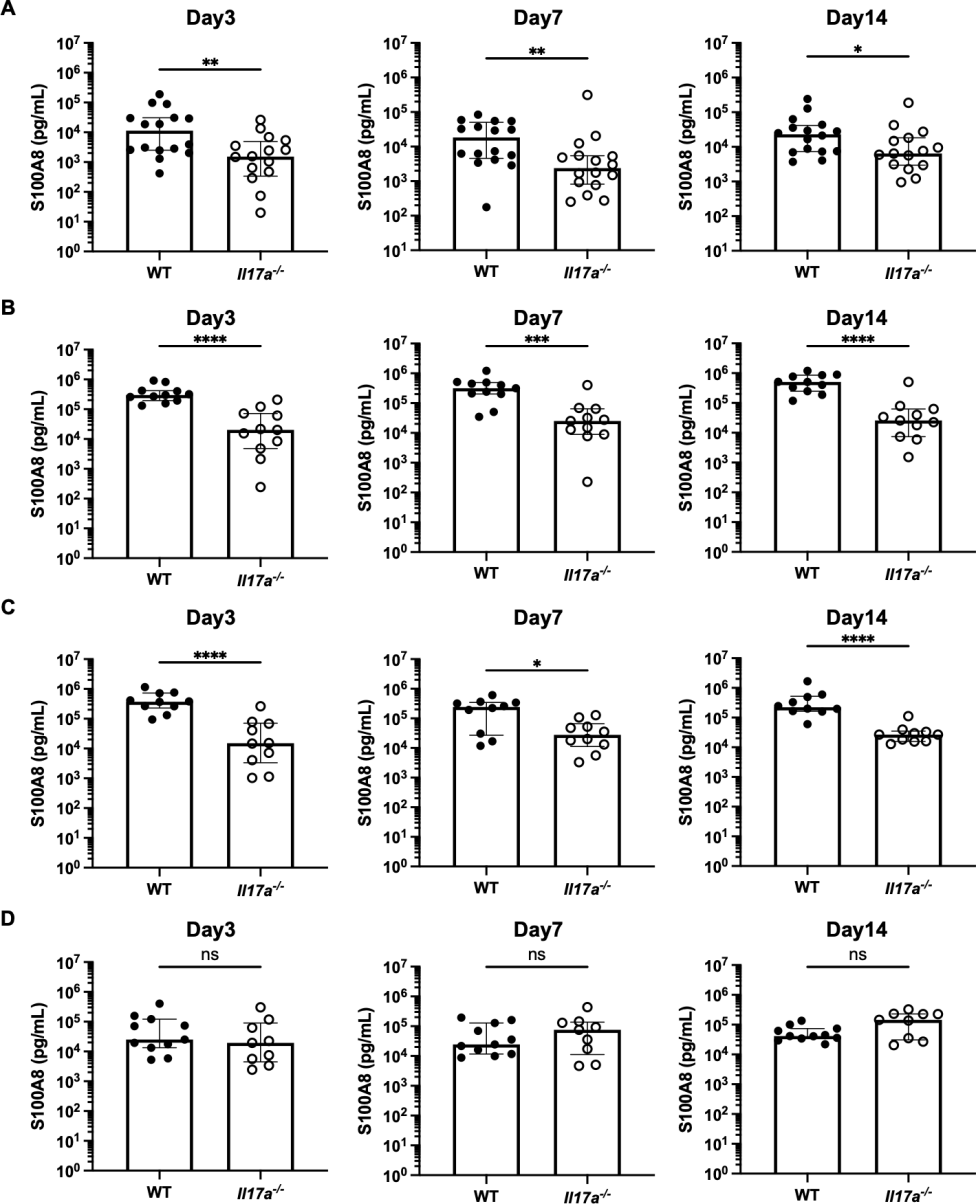
Concentration of the antimicrobial peptide S100A8 following inoculation with *C. auris* AR 0382 (**A**), AR 0383 (**B**), AR 0385 (**C**), and AR 0381 (**D**) in the vaginal lavage. S100A8 concentration in the vaginal lavage is presented in pg/mL. All results are based on at least three independent experiments and are expressed as mean ± standard error of the mean. ns, not significant; *P < 0.05, **P < 0.01, ***P < 0.001, ****P < 0.0001.

**Fig 4:**
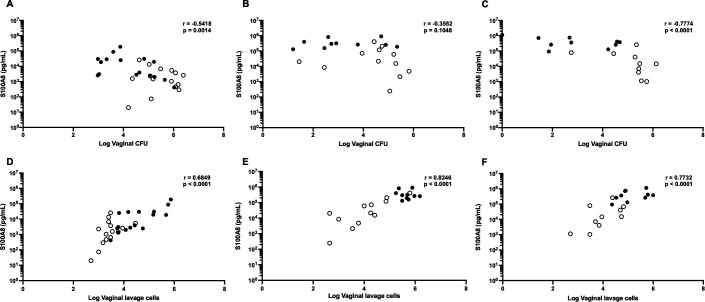
Correlations between S100A8 concentration (pg/mL) and vaginal fungal burden after inoculation with *Candida auris* AR 0382 (**A**), AR 0383 (**C**), and AR 0385 (**E**) and between S100A8 concentration and the number of inflammatory cells after inoculation with AR 0382 (**B**), AR 0383 (**D**), and AR 0385 (**F**). S100A8 concentration is shown as pg/mL, vaginal fungal burden is shown as log (CFU/100 µL), and the number of inflammatory cells is shown as log (cell number/100 µL) in the correlation plots. Results from at least three independent experiments were pooled for analysis. Filled circles and open circles represent samples from wild-type and *Il17a^−/−^* mice, respectively. “r” denotes Spearman’s rank correlation coefficient.

In addition to vaginal fungal colonization and inflammatory cell infiltration, we assessed the secretion of the antimicrobial peptide S100A8 in vaginal lavage fluid at 3, 7, and 14 days after inoculation with each *C. auris* strain. Vaginal S100A8 concentrations following inoculation with AR 0382, AR 0383, and AR 0385 were significantly higher in WT mice than in *Il17a^−/−^* mice throughout the experimental period (Fig. 3A through C). In contrast, no significant differences were observed between the two groups after AR 0381 inoculation (Fig. 3D). We then examined the correlation between fungal burden or vaginal inflammatory cell numbers and S100A8 concentration. Regarding the correlation between fungal burden and S100A8 concentration, there were significant negative correlations between these two factors in mice infected by AR 0382, AR 0383, and AR 0385 (Fig. 4A, C, and E). Additionally, there were significant positive correlations between the number of vaginal inflammatory cells and S100A8 concentrations in these groups (Fig. 4B, D, and F). Taken together, these results indicate that vaginal secretion of the antimicrobial peptide S100A8 occurs alongside inflammatory cell infiltration and contributes to the effective clearance of vaginal *C. auris*.

### Less neutrophil infiltration in the vagina after *C. auris* infection in *Il17a^−/−^* mice

**Fig 5:**
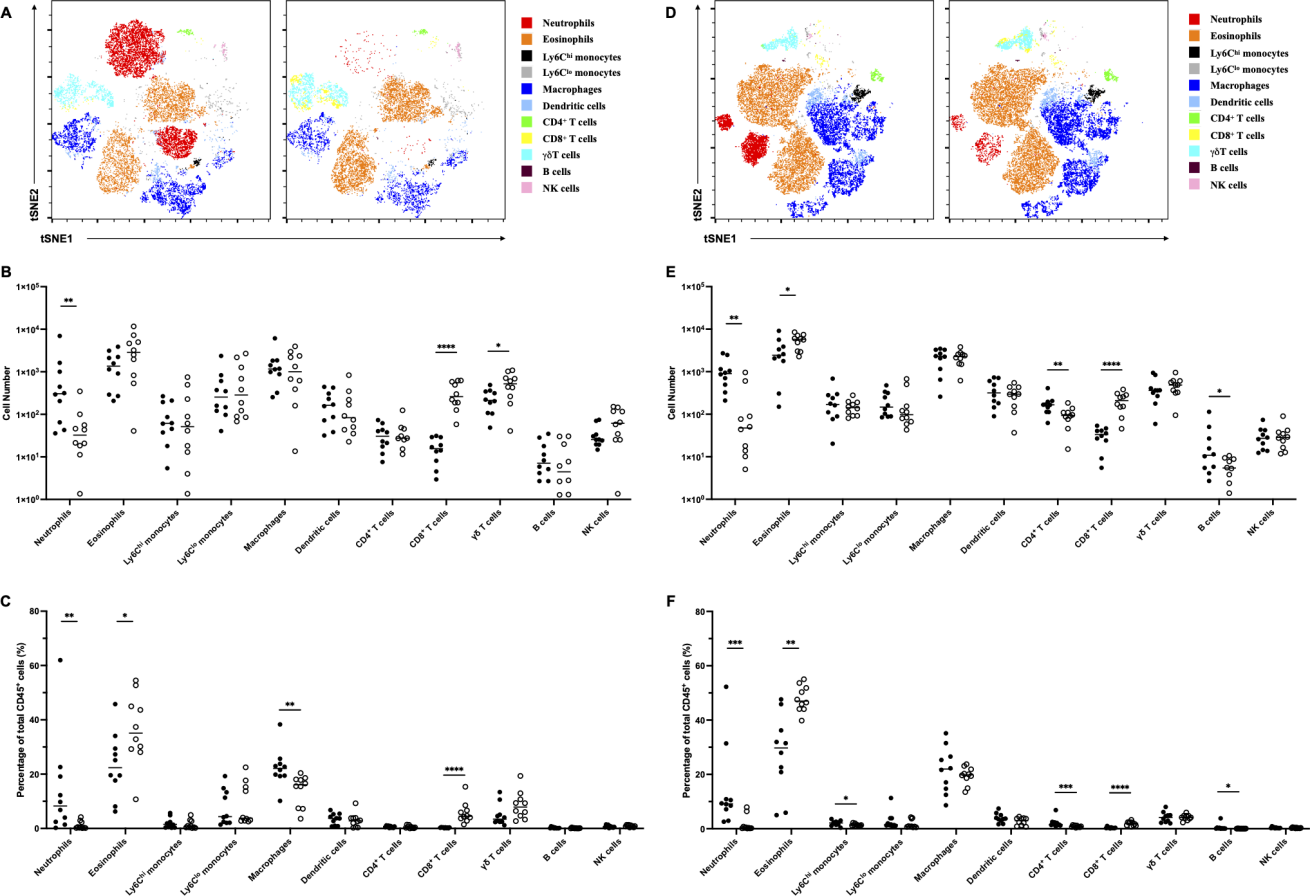
tSNE plots and quantification (cell number and percentage of total CD45^+^ cells) of vaginal immune cells at 3 days (**A–C**) or 7 days (**D–F**) after *C. auris* AR 0382 inoculation. Results from three independent experiments were pooled for analysis. Representative concatenated data of vaginal immune cells from one experiment are shown in the tSNE plots. *P < 0.05, **P < 0.01, ***P < 0.001, ****P < 0.0001.

Based on the above results, we further evaluated immune cell populations in the vagina tissue by flow cytometry at 3 and 7 days after *C. auris* AR 0382 inoculation (the gating strategy was shown in Fig. S3). Three days post-inoculation, a statistically significant increase in neutrophil numbers was observed in WT mice (Fig. 5A through C). In contrast, a significant increase in CD8^+^ T cells and γδ T cells was observed in *Il17a^−/−^* mice (Fig. 5B and C). At 7 days post-inoculation, neutrophil numbers remained higher in WT mice (Fig. 5D through F). Significant differences between the two groups were also found in eosinophils, CD4^+^ T cells, CD8^+^ T cells, γδ T cells, and B cells, although the absolute differences in lymphocyte numbers were relatively small (Fig. 5E). A similar trend in neutrophil and CD8^+^ T cell responses was also observed in mice inoculated with AR 0385 (Fig. S4). These results suggest that vaginal immune cells, particularly neutrophils, are significantly induced in WT mice following *C. auris* infection, potentially contributing to the host defense against vaginal *C. auris* colonization.

### Histopathological analysis showed less neutrophil infiltration and greater *C. auris* colonization in *Il17a^−/−^* mice

**Fig 6:**
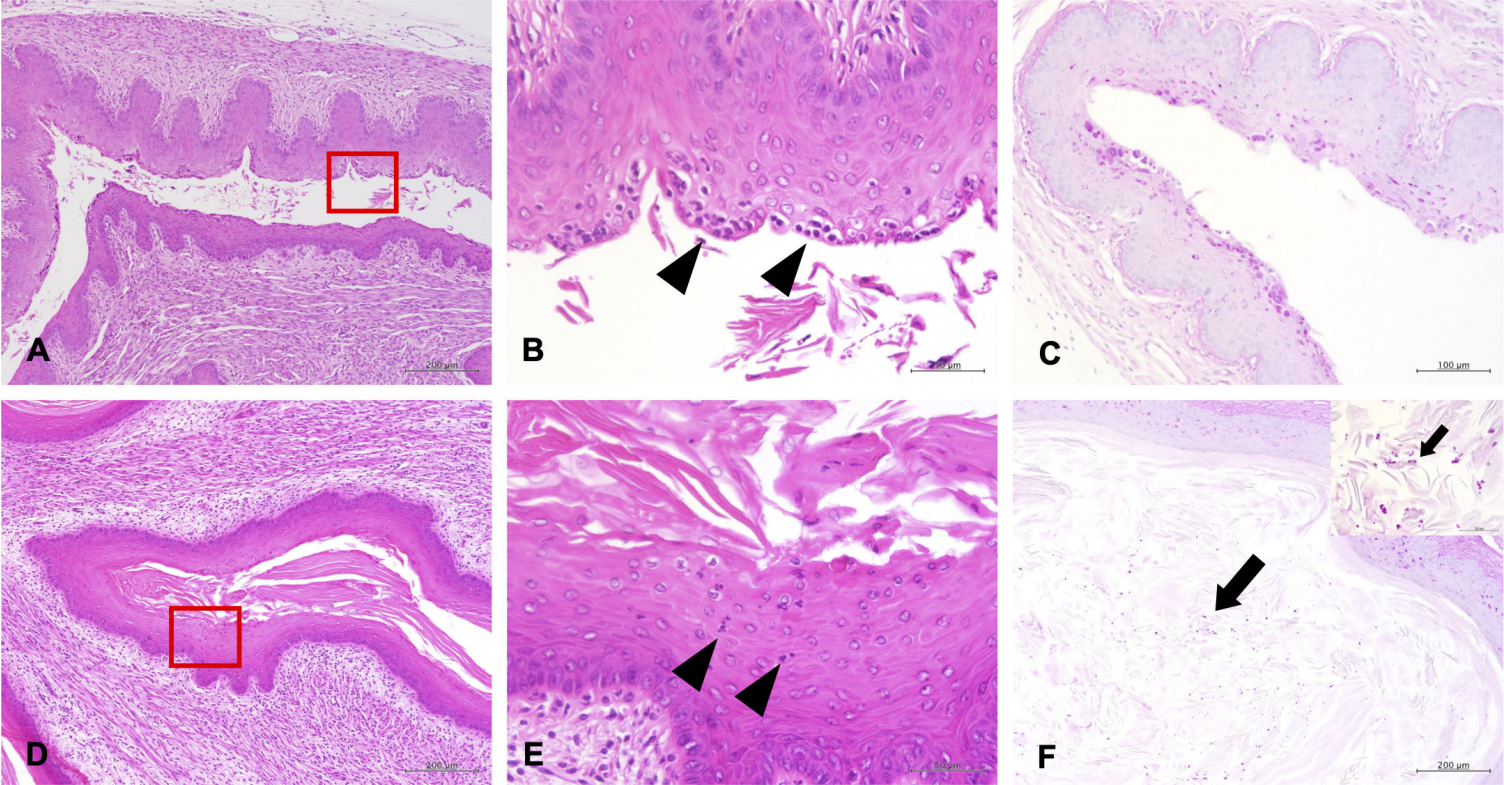
Histopathological analysis of vaginal tissues from mice at 7 days post-inoculation with *Candida auris*. Hematoxylin and eosin staining (**A, B**) and periodic acid-Schiff staining (**C**) in wild-type mice; hematoxylin and eosin staining (**D, E**); and periodic acid-Schiff staining in *Il17a^−/−^* mice (**F**). Arrows and arrowheads indicate yeast colonization in the vagina and neutrophil infiltration into vaginal epidermis, respectively. Original magnification of each figure is as follows: (**A, D, F**) ×100, (**B, E**) ×400, (**C**) ×200, (F inset [hyperview]) ×600. Panels **B** and **E **show the hyperview of the red grid inside panels **A **and **D**, respectively.

Given the results of fungal burden and inflammatory cell infiltration, we conducted histopathological analysis of vaginal tissues at 3 and 7 days after *C. auris* AR 0382 inoculation. In WT mice, no evident *C. auris* colonization was observed at either time point (Fig. 6A through C; Fig. S2A through C). In contrast, neutrophil infiltration into the vaginal epithelium was confirmed at both 3 and 7 days post-infection, consistent with the flow cytometry findings. However, *Il17a^−/−^* mice showed no significant neutrophil infiltration after inoculation (Fig. 6D and E; Fig. S2D and E). Instead, pronounced hyperkeratosis of the vaginal epithelium, accompanied by persistent fungal colonization, was observed (Fig. 6D through F; Fig. S2D through F). Collectively, these histopathological results confirm a higher vaginal fungal burden and reduced neutrophil infiltration in *Il17a^−/−^* mice, supporting the findings from vaginal fungal burden and flow cytometry analyses.

### Greater *C. auris* adhesion to vaginal cells notably under microaerobic condition

**Fig 7:**
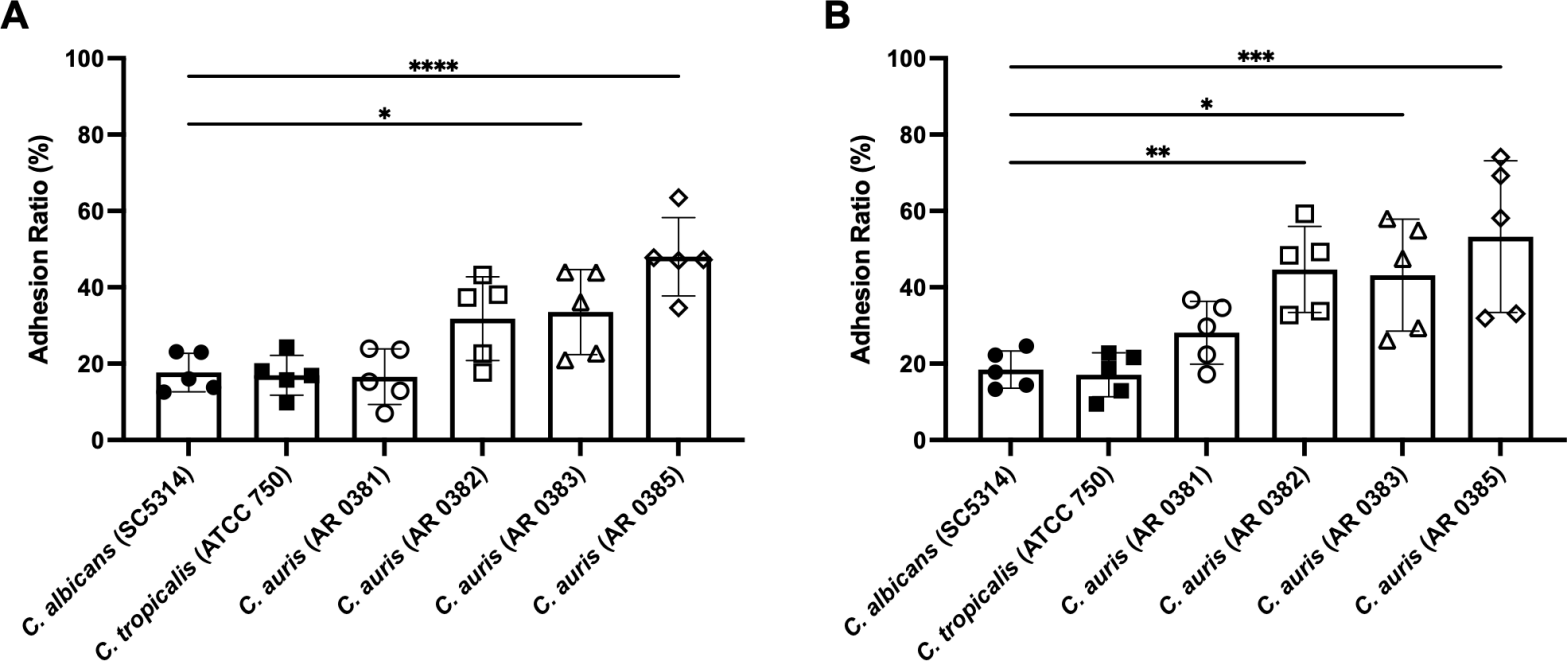
Adhesion ratios of various *Candida* species under aerobic (**A**) and microaerobic (**B**) conditions, calculated from CFU counts, are shown. All results are expressed as mean ± standard deviation from five independent experiments. Each symbol represents the mean of three replicates in each experiment. One-way ANOVA and *post hoc* analysis were performed using Dunnett’s multiple comparisons test, with *C. albicans* SC5314 as the control. *P < 0.05, **P < 0.01, ***P < 0.001, ****P < 0.0001.

Based on the *in vivo* results, we further evaluated the adhesive capacity of *C. auris* to vaginal epithelial cells in comparison with *C. albicans* and *C. tropicalis* (selected based on adhesive capability under microaerobic condition, capability of hyphal formation and vaginal colonization [19, 31]). Under aerobic conditions, certain *C. auris* strains exhibited significantly higher adhesive capacity than *C. albicans*, while no significant differences were observed between *C. albicans*, *C. tropicalis*, and *C. auris* AR 0381 (Fig. 7A). In contrast, under microaerobic conditions simulating the vaginal environment (28, 29), *C. auris* clade I, III, and IV strains showed significantly greater adhesion than *C. albicans* (Fig. 7B). Meanwhile, the adhesion of clade II *C. auris* AR 0381 remained comparable to that of *C. albicans*. These results suggest that *C. auris*, particularly clades I, III, and IV, possesses enhanced affinity for vaginal epithelial cells—especially under vaginal-like conditions—further supporting the *in vivo* findings of increased colonization in *Il17a^−/−^* mice.

## DISCUSSION

Vulvovaginal candidiasis (VVC) is one of the major forms of superficial candidiasis, and although *C. albicans* is the most common pathogen, all *Candida* species can potentially cause VVC in humans (7–10). *Candida auris* was originally isolated from the ear discharge of a patient without invasive candidiasis. However, it is now recognized as an emerging fungus capable of long-term colonization in the environment, as well as on human skin and mucosal membranes (11–15). It has been hypothesized that *C. auris* may colonize the human vagina, leading to prolonged VVC, although *C. auris* VVC is a rare disease and not consistently seen in clinical settings. However, no reports to date have described the relationship or associated factors between *C. auris* and VVC due to its low incidence rate. In our study, *C. auris*, particularly clades I, III, and IV, induced persistent vaginal colonization with reduced inflammatory cell infiltration under IL-17A-deficient conditions. This persistent colonization was associated with decreased secretion of the antimicrobial peptide S100A8 and reduced neutrophil infiltration in the vaginal tissue. Furthermore, *C. auris* exhibited greater adhesion to vaginal epithelial cells than *C. albicans*, especially under microaerobic conditions mimicking the vaginal environment. To the best of our knowledge, this is the first report demonstrating a strong association between *C. auris* and VVC, particularly under immunosuppressive conditions, such as IL-17A deficiency.

The analysis of vaginal fungal burdens revealed that colonization gradually decreased over time in WT mice. In contrast, *C. auris,* particularly clades I, III, and IV, persisted for a prolonged duration in *Il17a^−/−^* mice. Additionally, the recruitment of inflammatory cells into vaginal lavages was significantly lower in *Il17a^−/−^* mice. Previous reports have shown that *C. auris* can colonize the vaginas of WT mice. However, colonization typically decreased or was eliminated by 7 days post-inoculation, coinciding with inflammatory cell infiltration (19). Another study also reported that early recruitment of vaginal inflammatory cells was associated with the clearance of colonization in a *C. albicans* VVC mouse model (30). Flow cytometry analysis in our study further revealed significant differences in neutrophil numbers between WT and *Il17a^−/−^* mice following vaginal *C. auris* infection. IL-17A has previously been shown to be closely linked to neutrophil-mediated inflammation and host defense in *C. auris* skin infections (32–34). Thus, our findings suggest that IL-17A also plays a crucial role in the host defense against vaginal *C. auris*, likely contributing to its clearance via neutrophil recruitment.

In terms of immune cell populations involved in *C. auris* colonization, although our flow cytometry data showed significantly higher neutrophil numbers in WT mice, CD8^+^ T cell and γδ T cell counts also differed between the two groups. These cells may increase compensatorily and play a role in the defense response against *C. auris* under IL-17A-deficient conditions. Previous studies have shown that CD8^+^ T cells and γδ T cells are involved in *C. albicans* VVC and infections caused by other *Candida* species (30, 35–37). However, it has also been reported that CD8^+^ T cells and γδ T cells do not contribute to dermal *C. auris* colonization in mouse models (38). It is therefore hypothesized that immune defense mechanisms against *C. auris* may differ between the vagina and skin, although further studies are required to elucidate the exact mechanisms.

The analysis of antimicrobial peptide S100A8 in vaginal lavages showed that its concentration was significantly lower in *Il17a^−/−^* mice infected with *C. auris* clades I, III, and IV compared with that in WT mice. Previous studies have described a strong association between S100A8 and neutrophil recruitment (39–42). Additionally, our earlier research on *C. albicans* vulvovaginal candidiasis demonstrated that S100A8 concentration was positively correlated with vaginal inflammatory cell infiltration (30). In the present study, significant positive correlations were confirmed between S100A8 levels and vaginal inflammatory cell infiltration three days after *C. auris* inoculation. Moreover, significant negative correlations were observed between S100A8 concentration and vaginal fungal burden. In contrast, our results for AR 0381 (clade II) showed no significant differences in S100A8 levels between WT and *Il17a^−/−^* mice. Correspondingly, differences in vaginal fungal burden and inflammatory cell numbers were also minimal. These findings suggest that the vaginal defense mechanisms against clade II *C. auris* strains may differ from those against other clades. This variation may be due to clade-specific characteristics of *C. auris*, as clades I, III, and IV have been primarily associated with invasive candidiasis, whereas clade II strains are more commonly isolated from ear discharges and linked to non-invasive infections (11–15, 43). Taken together, these results suggest that early S100A8 secretion, along with inflammatory cell infiltration in the vagina, may be essential for the effective clearance of *C. auris* strains from clades other than clade II.

The *in vitro* adhesion assay using vaginal epithelial cells showed that *C. auris*, particularly clade I, III, and IV strains, exhibited greater adhesion under microaerobic conditions compared with *C. albicans*. No significant differences were observed between the *C. auris* clade II strain and *C. albicans* under either aerobic or microaerobic conditions. These results were consistent with our *in vivo* findings. It has been reported that the vaginal environment is microaerobic, characterized by low oxygen levels (approximately 3%–5%) and elevated carbon dioxide concentrations (approximately 5%–7%) (28, 29). Our *in vitro* experiments, conducted under these physiological conditions, clearly demonstrated enhanced adhesion of *C. auris* in a microaerobic environment mimicking the vagina. These findings correlate with our *in vivo* results and provide novel insight into the clade-specific phenotypes of *C. auris*.

There are several limitations to our study. First, we used only one strain from each *C. auris* clade. Therefore, it is possible that other strains within each clade could produce different results. However, our findings clearly showed a consistent trend among clade I, III, and IV strains, which was distinct from that of clade II. Thus, it is expected that strains from clades II, III, and IV may exhibit similar phenotypes in the mouse VVC model, although further investigation is needed to confirm this assumption. Second, our histopathological analysis revealed a fungal colonization in *Il17a^−/−^* mice, along with hyperkeratosis of the vaginal epithelium. However, the significance of these epithelial changes remains unclear. WT mice showed no such histological changes following *C. auris* infection, suggesting that this phenomenon may be specific to IL-17A deficiency. A previous study reported gastric hyperkeratosis in *Il17a^−/−^* mice with *C. albicans* oropharyngeal candidiasis (44). Based on this finding, hyperkeratosis may represent a reactive change in response to *Candida* infections. However, the underlying mechanisms have yet to be elucidated. Third, although we performed *in vitro* adhesion assay under microaerobic conditions, we only assessed adhesion after 2 h. Since VVC is typically a chronic infection, longer-term adhesion assays would be necessary to fully understand the mechanisms of persistent colonization. However, conducting prolonged adhesion studies under microaerobic conditions is challenging, as vaginal epithelial cells might not survive for longer in hypoxic *in vitro* environments, which was different from human bodies in terms of blood and nutrient supply. Therefore, alternative platforms—such as vagina-on-a-chip or organoid systems—will be required in future studies to evaluate long-term adhesive capability.

In conclusion, our results indicate that *C. auris* can establish persistent vaginal colonization accompanied by reduced induction of neutrophils and the antimicrobial peptide S100A8 under IL-17A-deficient conditions. Furthermore, *C. auris* clade I, III, and IV strains exhibited a unique phenotype of enhanced adhesion to vaginal epithelial cells under microaerobic conditions mimicking the vaginal environment. Our investigation suggests a strong association between *C. auris* and VVC in immunocompromised hosts, providing novel insight with potential clinical relevance.

## MATERIALS AND METHODS

### Mice

Animal experiments were conducted using age-matched, female, 6- to 8-week-old IL-17A-knockout (*Il17a^−/−^*) mice (kindly gifted from Prof. Iwakura, Tokyo University of Science) on a C57BL/6J background and corresponding wild-type (WT) C57BL/6J (not littermate) controls (45). Mice were bred and maintained under specific pathogen-free conditions at the National Institute of Infectious Diseases, Japan.

### *Candida auris* strains and preparation

For the mouse vulvovaginal candidiasis model, *C. auris* strains AR 0382 (clade I), AR 0381 (clade II), AR 0383 (clade III), and AR 0385 (clade IV) were obtained from the CDC’s AR Bank. Each strain was cultured on yeast extract peptone dextrose (YPD) agar at 30°C for 2–3 days. A single colony was then transferred to YPD broth and incubated at 30°C for 18–24 h. After incubation, cells were collected, washed, and resuspended in sterile phosphate-buffered saline (PBS) for use in experiments.

### Mouse vulvovaginal candidiasis model

The established mouse vulvovaginal candidiasis model was used as previously described (30). Briefly, 0.2 mg of β-estradiol 17-valerate (Nacalai Tesque, Kyoto, Japan) dissolved in 0.1 mL of sesame oil was subcutaneously administered to each mouse one week before *C. auris* inoculation. To maintain a pseudo-estrus state, β-estradiol 17-valerate was subsequently administered once weekly until the end of the experiments. Mice were then intravaginally inoculated with approximately 1.0 × 10^7^ colony-forming units (CFU) of prepared *C. auris* suspension to induce vulvovaginal candidiasis.

### Evaluation of vaginal fungal burden and infiltration of inflammatory cells

To evaluate fungal burden, vaginal lavage fluid was collected from each mouse under isoflurane anesthesia by gently flushing the vagina twice with 50 µL of sterile PBS per wash. The aspirated lavage fluid was serially diluted and plated on YPD agar supplemented with penicillin/streptomycin. Plates were incubated at 30°C for 48 h before counting fungal colonies. Vaginal inflammatory cells were stained with trypan blue and counted under a light microscope using a hemocytometer, a method reported to be consistent with Papanicolaou staining (30). The remaining lavage fluid was centrifuged and stored at −30°C for subsequent cytokine analysis.

### Histopathological analysis of vagina from infected mice

For histopathological analysis, vaginal tissues were collected from each mouse at 3 or 7 days post-inoculation, fixed in 10% neutral-buffered formalin, dehydrated in ethanol, and embedded in paraffin following standard procedures. Sections (4 µm thick) were mounted on glass slides and stained with hematoxylin and eosin or periodic acid-Schiff (PAS). Histological examinations were then performed using light microscopy.

### Evaluation of vaginal lavage S100A8 concentration

The concentration of the antimicrobial peptide S100A8 in vaginal lavage fluid was measured using a commercial S100A8 enzyme-linked immunosorbent assay (ELISA) kit (R&D Systems, Minneapolis, MN, USA), following the manufacturer’s instructions.

### Vagina immune cells isolation and flow cytometry analysis

Three or seven days after *C. auris* inoculation, each mouse was euthanized, and the vagina was carefully isolated, washed, and minced. The tissue was then incubated with 1.3 units/mL Liberase TL (Roche Applied Science, Penzberg, Germany) and 200 µg/mL DNase I (Roche Applied Science, Penzberg, Germany) at 37°C for 60 min with continuous rotation. Following enzymatic digestion, the tissue was gently homogenized and passed through a 70 µm nylon-mesh filter to obtain a single-cell suspension, and then, the resulting vaginal single cells were washed twice. Vaginal lavages were centrifuged and washed twice. Vaginal tissue and lavage cells are stained with the Live/Dead Fixable Blue Dead Cell Stain Kit (Invitrogen, California, USA) at room temperature for 10 min. After dead cell staining, cells were incubated with various fluorochrome-conjugated antibodies (listed in Table 1) at 4°C for 45 min. Following staining, cells were washed and fixed with 4% paraformaldehyde at room temperature for 15 min. Immune cells were then quantified using an Aurora-CS flow cytometer (Cytek Biosciences, Fremont, CA, USA) with CountBright Absolute Counting Beads (Invitrogen, California, USA). Flow cytometric data were analyzed using FlowJo software, version 10.10 (Tree Star, Inc., Ashland, OR, USA).

**TABLE 1:** The antibodies used in this study^*a*^

Antigen	Clone	Conjugated fluorochrome	Supplier
CD45	30-F11	BUV395	BD
CD11c	HL3	BUV496	BD
Siglec-F	1RNM44N	BUV615	Invitrogen
CD11c	N418	BUV615	Invitrogen
I-A/I-E	M5/114.15.2	BUV805	BD
CD4	GK1.5	BV421	BioLegend
Siglec-F	E50-2440	BV480	BD
Ly6C	HK1.4	BV605	BioLegend
Ly6G	1A8	BV750	BD
CD8a	53-6.7	FITC	BioLegend
TCRγδ	GL3	APC	BioLegend
B220	RA3-6B2	AF700	BioLegend
F4/80	BM8	PE	BioLegend
CD11b	M1/70	PE Dazzle594	BioLegend
CD3	17/A2	PE/Fire700	BioLegend
NK1.1	PK136	APC-eFluor780	Invitrogen
CD16/32	93	Fc Blocking	BioLegend

^*a*^ BUV, brilliant ultraviolet; BV, brilliant violet; FITC, fluorescein isothiocyanate; PE, phycoerythrin; APC, allophycocyanin; AF700, Alexa Fluor 700.

### *Candida* species adhesion assay to vagina epithelial cell line VK2/E6E7

VK2/E6E7, a human vaginal epithelial cell line (ATCC, CRL-2616), was cultured in keratinocyte serum-free medium (K-SFM; Invitrogen, CA, USA) on a 90 mm dish at 37°C in a 5% CO_2_ atmosphere until confluency. The cells were then detached using trypsin/EDTA solution (Fujifilm Wako Pure Chemical Corporation, Osaka, Japan), washed, and seeded into 24-well plates. The seeded VK2/E6E7 cells were incubated in K-SFM for 7 days to allow formation of a confluent monolayer. *C. albicans* SC5314, *C. tropicalis* ATCC 750, and *C. auris* strains AR 0381, AR 0382, AR 0383, and AR 0385 were grown on YPD agar at 37°C for 2–3 days under either aerobic or microaerobic conditions (5% O_2_/5% CO_2_). A single colony was then inoculated into yeast nitrogen base (BD Difco, USA) with 2% glucose broth and incubated at 37°C with shaking under aerobic conditions or without shaking under microaerobic conditions for 18–24 h. After incubation, *Candida* cells were collected, washed, and resuspended in Roswell Park Memorial Institute (RPMI) medium with 3-morpholinopropanesulfonic acid and 1% glucose. Approximately 5.0 × 10^4^ cells of each *Candida* strain were inoculated onto each well containing the epithelial cell monolayer and incubated for 2 h under either aerobic (5% CO_2_) or microaerobic (5% O_2_/5% CO_2_ conditions). After incubation, wells were gently washed three times with sterile PBS, and adherent cells were detached using trypsin/EDTA. Both the initial inoculum and the detached (adherent) cells from each well were plated onto YPD agar and incubated at 30°C for 24–48 h for colony enumeration. The adhesion ratio (%) was calculated as [CFU of stripped cells]/[CFU of inoculum] × 100.

### Statistical analysis

The continuous variables of the two groups were compared using the Student *t*-test, assuming equal variance. If the standard deviations differed between the groups, the Mann–Whitney *U*-test was applied. For comparisons among three or more groups in the *in vitro* assay, one-way analysis of variance (ANOVA) was conducted, followed by *post hoc* analysis using Dunnett’s multiple comparisons test. Correlation analysis was performed using Spearman’s rank correlation coefficient. A *P*-value of less than 0.05 from the two-tailed test was considered significant for all tests. Statistical analyses were performed using GraphPad Prism, version 10 (GraphPad Software, La Jolla, CA, USA).

## SUPPLEMENTAL MATERIAL

Fig. S1 msphere.00446-25-s0001.docx Representative flow cytometry gating strategy of vaginal lavage in WT and *Il17a*^−/−^ mice.

Fig. S2 msphere.00446-25-s0002.docx Histopathological analysis of vaginal tissues from mice at 3 days post-inoculation with *C. auris*.

Fig. S3 msphere.00446-25-s0003.docx Representative flow cytometry gating strategy of vagina tissues.

Fig. S4 msphere.00446-25-s0004.docx Quantification of vaginal immune cells at 3 or 7 days after *C. auris* AR 0385 inoculation.

ASM does not own the copyrights to Supplemental Material that may be linked to, or accessed through, an article. The authors have granted ASM a non-exclusive, world-wide license to publish the Supplemental Material files. Please contact the corresponding author directly for reuse.
