# Supplementary figures and images for: Candida auris persists in the vaginal microaerobic niche in the absence of interleukin-17A
Source: mSphere. 2025 Oct 8;10(10):e00446-25. doi: 10.1128/msphere.00446-25 (PMC12570508; doi:10.1128/msphere.00446-25)

**Fig S1**

**
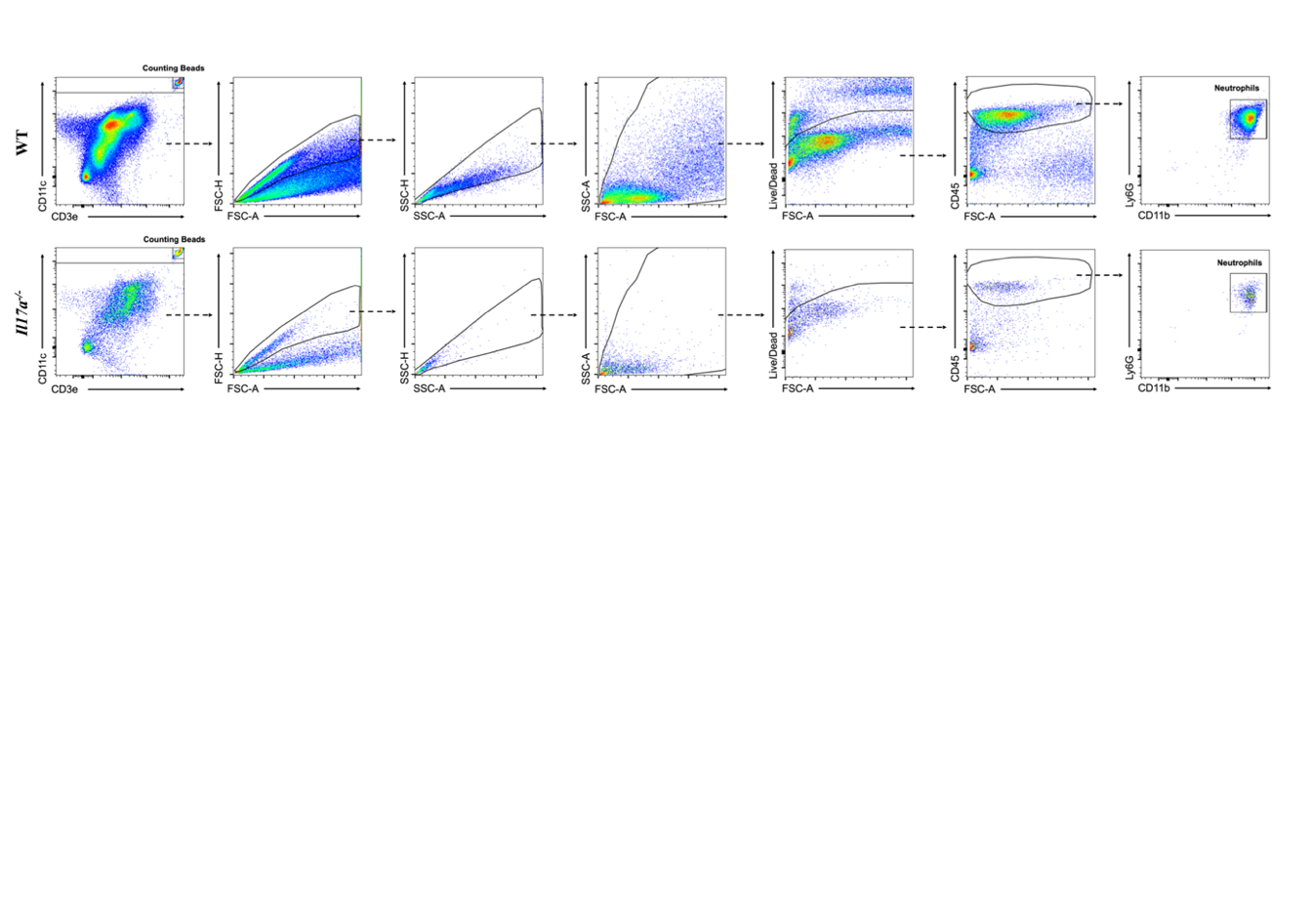
**

**Fig S1** Representative flow cytometry gating strategy of vaginal lavage in WT and *Il17a-/-* mice.

Supplement: Fig. S1 — Representative flow cytometry gating strategy of vaginal lavage in WT and Il17a−/− mice. [file msphere.00446-25-s0001.docx]
