# Supplementary material for: Candida auris persists in the vaginal microaerobic niche in the absence of interleukin-17A
Source: mSphere. 2025 Oct 8;10(10):e00446-25. doi: 10.1128/msphere.00446-25 (PMC12570508; doi:10.1128/msphere.00446-25)
Supplement: Fig. S2 — Histopathological analysis of vaginal tissues from mice at 3 days post-inoculation with C. auris. [file msphere.00446-25-s0002.docx]

**Fig S2**

**
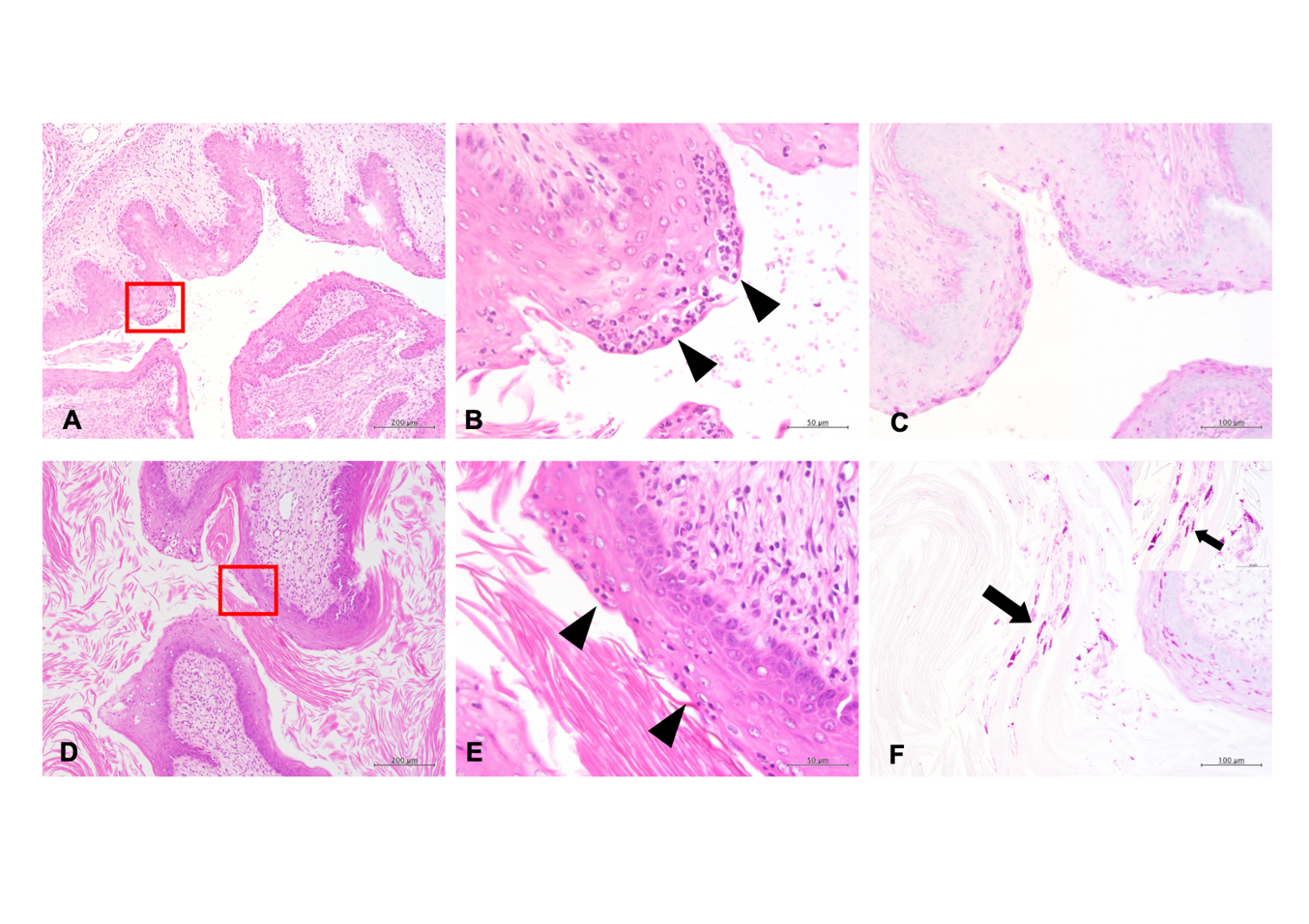
**

**Fig S2** Histopathological analysis of vaginal tissues from mice at 3 days post-inoculation with *Candida auris*. Hematoxylin and eosin staining (A, B) and periodic acid schiff staining (C) in wild-type mice; hematoxylin and eosin staining (D, E) and periodic acid schiff staining in *Il17a^-/-^* mice (F). Arrows and arrowheads indicate yeasts colonization in vagina and neutrophil infiltration into vaginal epidermis, respectively. Original magnification of each figure is as follows: (A, D, F) ×100, (B, E) ×400, (C, F) ×200, (F’: right upper hyperview) ×600. (B) and (E) show the hyperview of the red grid inside (A) and (D), respectively.
