# Supplementary material for: Candida auris persists in the vaginal microaerobic niche in the absence of interleukin-17A
Source: mSphere. 2025 Oct 8;10(10):e00446-25. doi: 10.1128/msphere.00446-25 (PMC12570508; doi:10.1128/msphere.00446-25)
Supplement: Fig. S3 — Representative flow cytometry gating strategy of vagina tissues. [file msphere.00446-25-s0003.docx]

**Fig S3**

**
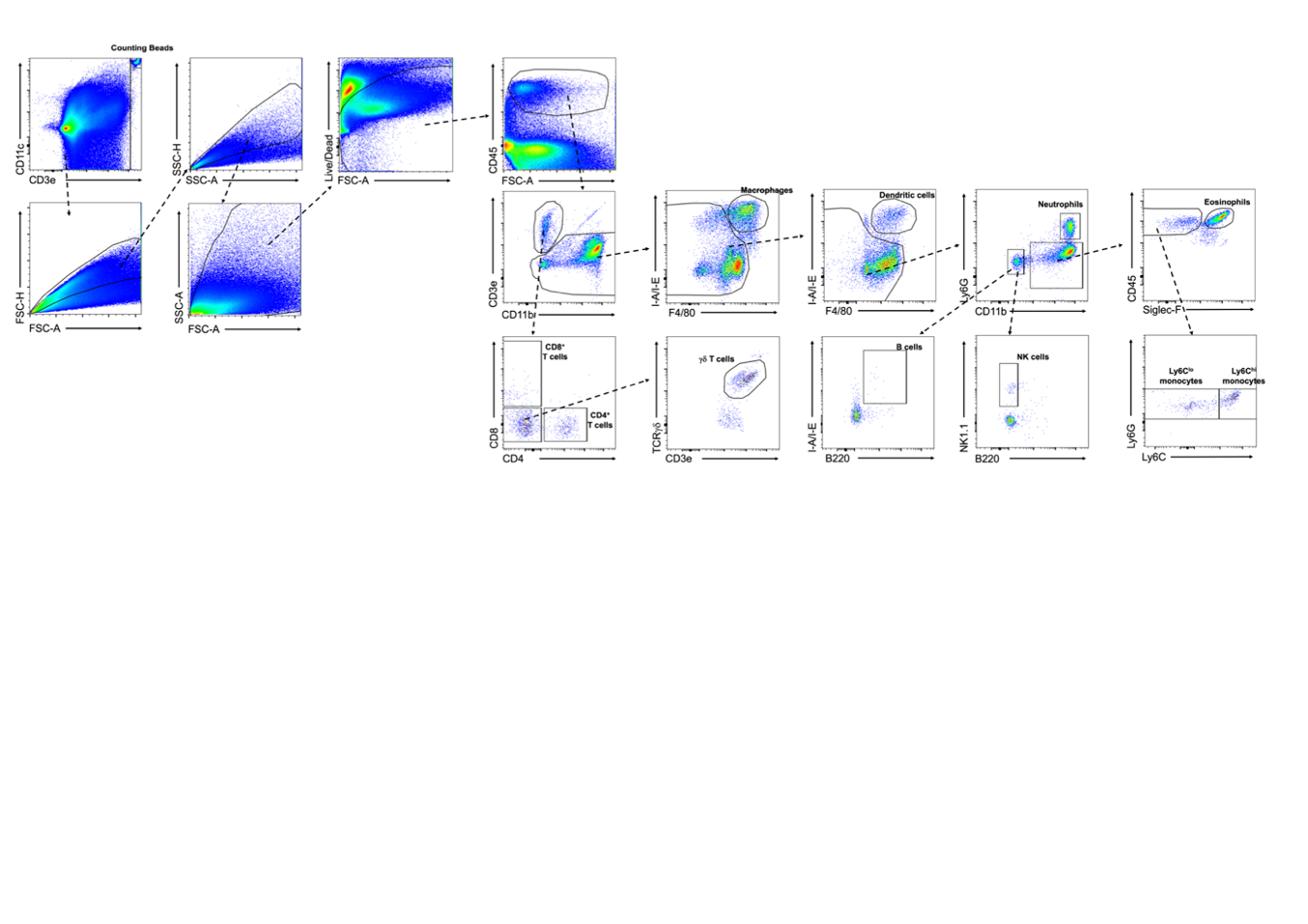
**

**Fig S3** Representative flow cytometry gating strategy of vagina tissues. Concatenated data of 4 WT mice were shown.
