# Supplementary material for: Candida auris persists in the vaginal microaerobic niche in the absence of interleukin-17A
Source: mSphere. 2025 Oct 8;10(10):e00446-25. doi: 10.1128/msphere.00446-25 (PMC12570508; doi:10.1128/msphere.00446-25)
Supplement: Fig. S4 — Quantification of vaginal immune cells at 3 or 7 days after C. auris AR 0385 inoculation. [file msphere.00446-25-s0004.docx]

**Fig S4**

**
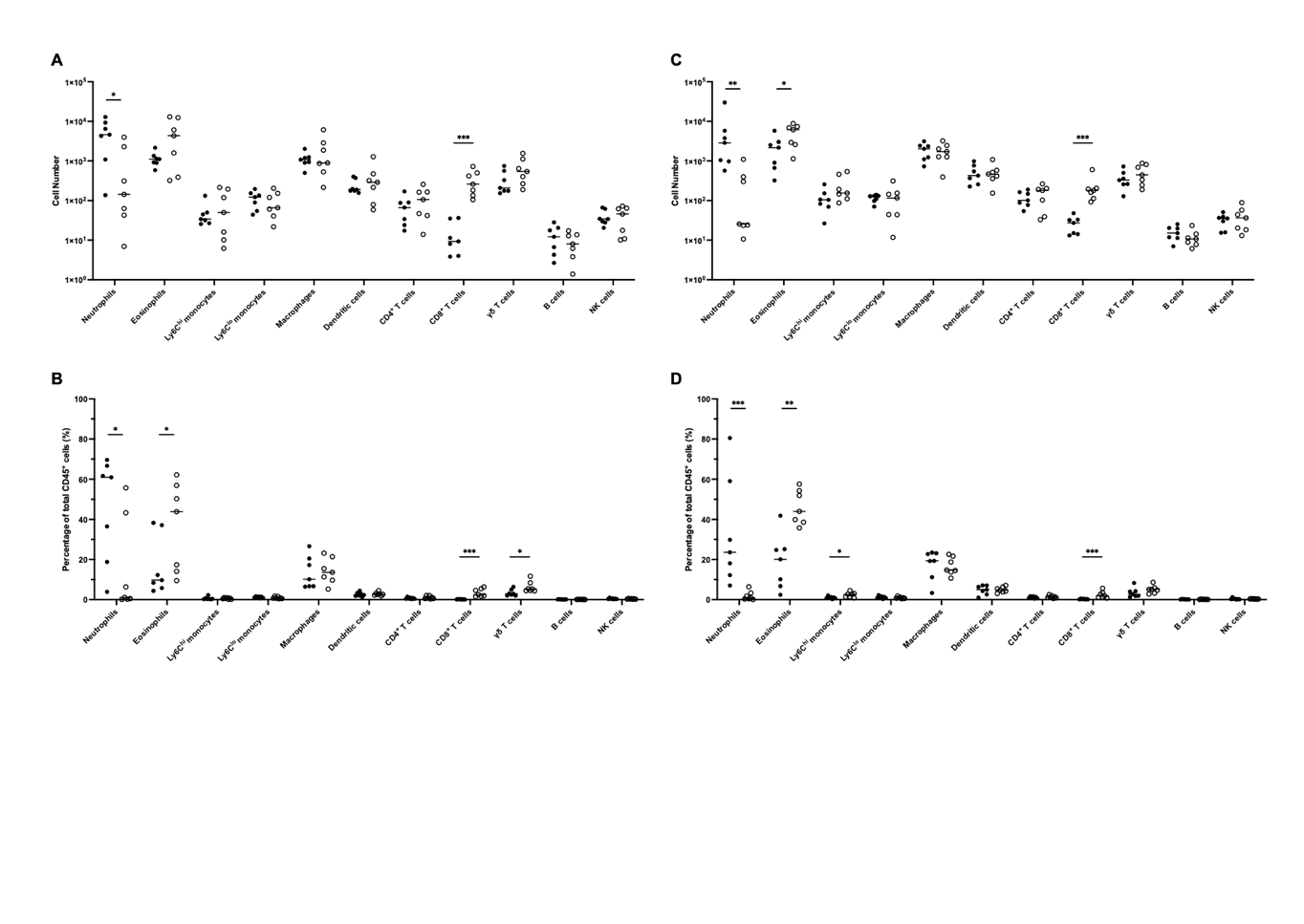
**

**Fig S4** Quantification (cell number and percentage of total CD45^+^ cells) of vaginal immune cells at 3 days (A, B) or 7 days (C, D) after *C. auris* AR 0385 inoculation. Results of two independent experiments were pooled for analysis. **p* < 0.05, ***p* < 0.01, ****p* < 0.001.
